# Digitally Controlled Piezoelectric Metamaterial for Low-Frequency and High-Efficiency Sound Absorption

**DOI:** 10.3390/ma18092102

**Published:** 2025-05-03

**Authors:** Xiaodong Zhang, Jing Nie, Jinhong He, Fengbin Lin, Yang Liu

**Affiliations:** 1 College of Mechanical Engineering and Automation, Huaqiao University, Xiamen 361021, China; 23014080068@stu.hqu.edu.cn (J.N.); 23014080029@stu.hqu.edu.cn (J.H.); 24014080034@stu.hqu.edu.cn (F.L.); 2 Jilin Jinheng Auto Parts Co., Ltd., Jilin 132000, China

**Keywords:** acoustic metamaterial, sound absorption, piezoelectric patch, resonant frequency, digital control

## Abstract

This study proposes a membrane-type metamaterial with digitally controlled piezoelectric actuation for low-frequency sound absorption applications. The hybrid structure integrates an aluminum membrane functionally bonded with programmable piezoelectric patches (PZTs) and a sealed air cavity. Two innovative control strategies—Resistance Enhancement and Resonance Enhancement—dynamically adjust circuit impedance to maximize electromechanical energy conversion efficiency, thereby optimizing absorption at targeted frequencies. These strategies are implemented via a real-time digital feedback system. A coupled piezoelectric-structural-acoustic model is established to characterize the system’s transfer function, with validation through both finite element simulations and impedance tube experiments. Numerical and experimental results demonstrate nearly complete absorption around the resonant frequency, and the bandwidth can be further broadened through multi-resonance superposition. Theoretical analysis confirms that the active control strategies simultaneously modulate the acoustic impedance components (resistance and reactance), thereby optimizing electromechanical energy conversion efficiency. This work establishes a novel active-control methodology for low-frequency and high-efficiency noise mitigation.

## 1. Introduction

There is an escalating prevalence of low-frequency noise pollution in industrial and urban environments, such as automotive engine compartments, air conditioning systems, subway stations, and industrial machinery rooms, where low-frequency noise (100–500 Hz) from rotating machinery, fluid dynamics, or structural vibrations poses significant health and environmental challenges. Traditional noise control methods utilize porous fibers, foams, and microperforated plates [1,2], rely on viscous-thermal dissipation mechanisms through submillimeter-scale pores, and exhibit frequency-dependent absorption coefficients that decline significantly in low frequency [3]. Therefore, manufacturing efficient, compact, low-frequency broadband absorbing materials poses a significant challenge.

Acoustic metamaterials, defined as artificial structures with subwavelength-scale geometries, enable unconventional sound manipulation beyond the capabilities of natural materials, such as a negative refractive index or subwavelength absorption [3,4,5]. As structural materials, acoustic metamaterials can manipulate sound by adjusting structures at deep subwavelength scales. Thus, well-engineered metamaterials can create low-frequency bandgaps without bulky modules for low-frequency sound isolation or reduction. For instance, spatial spiral design can produce considerable sound absorption effects at subwavelength thickness [6,7,8,9], while the metamaterials formed by the Helmholtz structures also confer commendable sound absorption performance at their resonance [10,11,12,13]. By varying the geometry and assembling metamaterials at different operating frequencies, this strategy could widen their sound absorption frequency range [14,15,16]. Moreover, perfect sound reduction performance can also be achieved by designing a thin membrane on top of air cavities [17,18,19]. The absorption frequency can be reduced by adhering small mass blocks to the membrane, resulting in a more compact structure with thicknesses much smaller than the operating wavelength. These research findings indicate promising potential for vibration and noise control, yet modifications in frequency range post-structural fixation present limitations as the operating frequency is inherently tied to physical geometry.

Multifield-coupled active metamaterials offer dynamic tunability through external field modulation [20,21]. Among these, piezoelectric materials stand out for their small size, light weight, and compatibility with electronic control circuits. Pioneering studies have implemented shunted piezoelectric networks for noise and vibration control [22,23,24,25]. Unlike passive acoustic materials (e.g., porous foams), which rely on inherent visco-thermal losses, piezoelectric metamaterials offer active tuning through piezoelectric coupling, enabling dynamic adjustment of absorption frequency without geometric modifications. Passive absorbers with piezoelectric membranes or patches can facilitate single or multi-modal sound insulation capabilities at tuned resonant frequencies controlled by different circuits, and their sound insulation frequency range can be varied by adjusting shunt circuit parameters [26,27,28]. Notably, Liao’s work [29] proposes an adaptive absorption method incorporating negative capacitors and inductors. However, its practical implementation is constrained by technical challenges in constructing simultaneous negative capacitor–inductor circuits, which often induce system instability, high costs, and complex debugging burdens [30,31]. To address these challenges, Wang et al. [32,33] introduced two active enhancement strategies—the resonance-amplifier and amplifier-resonance strategies—for improved low-frequency vibration attenuation. Subsequently, Wang et al. [34] implemented these active control mechanisms in a metamaterial beam using digital control technology, achieving multi-modal vibration suppression. Meanwhile, recent advancements in the digital control of piezoelectric shunts have expanded metamaterials’ tunability [35,36,37,38,39]. For instance, Yan et al. [40] provided a comprehensive review of shunt damping technologies, highlighting the importance of impedance modulation in achieving broadband noise suppression. Marakakis et al. [41] systematically compared various piezoelectric shunting strategies, demonstrating the superiority of active enhancement methods in vibration control. Additionally, Zheng et al. [42] conducted both analytical and experimental investigations on a piezoelectric meta-shell shunted with digital circuits, achieving programmable multiple bandgaps and effectively suppressing structural vibration and sound radiation.

In summary, acoustic metamaterials shunted with piezoelectric materials demonstrate significant potential for efficient and adjustable sound absorption. However, related research remains insufficient. Inspired by these advancements, this paper introduces a membrane-type metamaterial shunted with digitally controlled piezoelectric patches (PZTs). By employing a novel digital control strategy, the proposed metamaterial achieves adjustable, near-perfect sound absorption at programmable frequencies, offering a promising solution for broadband noise control.

The subsequent sections are organized as follows: Section 2 details the design and theoretical model of the proposed metamaterial, including its structural configuration and governing equations. Section 3 presents an acoustic coupling analysis model based on equivalent medium theory and finite element methods, elucidating sound absorption mechanisms through the relationship between resistance and acoustic factors. Section 4 employs numerical analysis and experimental validation to investigate sound absorption performance, providing insights into the underlying physical mechanisms. Finally, Section 5 summarizes the key findings from both theoretical and experimental results, highlighting the practical implications and future research directions of this work.

## 2. Physical Model and Theory

### 2.1. Design of the Metamaterial

This section first introduces a hybrid structure combining an aluminum membrane and piezoelectric patches (PZTs), termed a ‘membrane-type metamaterial’ here. This design leverages the vibration of a membrane and PZTs, as well as air cavity resonance to achieve low-frequency sound absorption. The design principles and active control circuits are then elaborated. Subsequently, effective medium theory is employed to derive the effective bending parameters of the membrane shunted with the circuit, establishing analytical models for piezoelectric-structural-acoustic interactions that incorporate the active enhancement control mechanism. Ultimately, a finite element analysis model is constructed based on these equivalent parameters, enabling detailed numerical simulations of the metamaterial’s vibration and sound absorption performance.

**Figure 1:**
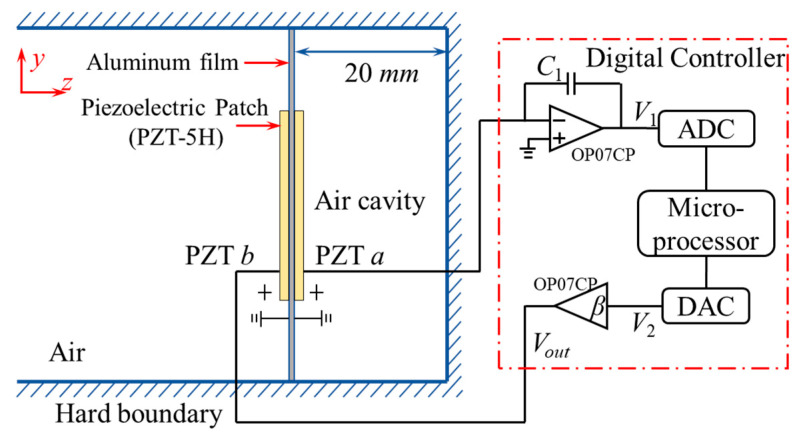
Sketch of the proposed membrane-type metamaterial shunted with a digital circuit.

Figure 1 presents the conceptual design of the proposed acoustic metamaterial and its corresponding digital control circuit. The metamaterial consists of two key components: an aluminum membrane with PZTs bonded on each side, serving as a sensing unit and actuating unit, respectively; and a cavity with a depth of 20 mm behind the composite membrane. The PZTs are polarized in opposite directions along the *z*-axis, interfacing with a digital controller for active piezoelectric shunt control. The control circuit comprises a charge amplifier, an analog-to-digital converter (ADC), a microprocessor, a digital-to-analog converter (DAC), and a proportional amplifier. Mechanical stress from PZT *a* is converted into a voltage signal *V*_1_ through the charge amplifier, and further digitized by the ADC. Using these digital signals, the microprocessor calculates the output signal *V*_2_ in real-time based on a specific transfer function. The DAC then converts the digital signal into an analog voltage signal *V*_2_, which is amplified by a factor of *β* and applied to PZT *b* via the proportional amplifier, achieving active piezoelectric shunt control according to the designed transfer function. The geometric and material parameters of the proposed metamaterial are listed in Table 1 and Table 2.

### 2.2. Design of the Transfer Function

During acoustic simulation, the PZTs bonded to the membrane undergo deformation, generating strain that drives the piezoelectric effect. This necessitates the inclusion of strain effects in our analysis. Accordingly, the membrane region bonded with the PZT is modeled as a thin flexible plate, and its equivalent elastic modulus is calculated using classical laminated plate theory to derive the transfer function required by the digital controller.

As shown in Figure 1, we define the *z*-axis as perpendicular to the electrode surface on the PZT (parallel to the aluminum membrane). The constitutive equation of the PZT is [26] as follows:

(1) $$\left\{\begin{array}{l} S_{1}=s_{11}^{E}T_{1}+s_{12}^{E}T_{2}+d_{31}E_{3},\\ S_{2}=s_{12}^{E}T_{1}+s_{11}^{E}T_{2}+d_{31}E_{3},\\ S_{6}=2\left(s_{11}^{E}-s_{12}^{E}\right)T_{6},\\ D_{3}=d_{31}T_{1}+d_{31}T_{2}+\varepsilon_{33}^{T}E_{3}, \end{array}\right.$$

where *S*_1_, *T*_1_, *S*_2_, and *T*_2_ denote normal strains and stresses along *x* and *y* axes; *S*_6_ and *T*_6_ represent shear strains and stresses perpendicular to the *x* axis and along the *y* axis, respectively. *D*_3_ denotes the electric displacement on the electrode; *E*_3_ represents the electric field intensity; $S_{11}^{E}$ and $S_{12}^{E}$ symbolize flexibility coefficients of a piezoelectric material under constant field strength; $\varepsilon_{33}^{T}$ represents the permittivity of the piezoelectric material under static strain; and *d*_31_ is the piezoelectric constant.

The piezoelectric coupling effect, described by Equation (1), establishes a bidirectional energy conversion between mechanical deformation of the membrane and electrical signals in the PZTs. This mechanism is distinct from electromagnetic coupling as it relies on electrostatic interactions within the piezoelectric material rather than magnetic fields, making it suitable for low-frequency acoustic applications.

PZT *a*, serving as a sensor unit, equates ground, hence its field intensity *E*_3_ is zero, which simplifies its piezoelectric equation. The constitutive equation of PZT *a* can be expressed as follows:

(2) $$\left\{\begin{array}{c} S_{1}^{a}\\ S_{2}^{a}\\ S_{6}^{a} \end{array}\right\}=\left[\begin{array}{ccc} S_{11}^{E} & S_{12}^{E} & 0\\ S_{12}^{E} & S_{11}^{E} & 0\\ 0 & 0 & 2(S_{11}^{E}-S_{12}^{E}) \end{array}\right]\left\{\begin{array}{c} T_{1}\\ T_{2}\\ T_{6} \end{array}\right\}$$

Obviously, Equation (2) resembles the standard stress–strain formula for isotropic plates:

(3) $$\left\{\begin{array}{c} S_{1}\\ S_{2}\\ S_{6} \end{array}\right\}=\left[\begin{array}{ccc} \frac{1}{E_{p}} & -\frac{v_{p}}{E_{p}} & 0\\ -\frac{v_{p}}{E_{p}} & \frac{1}{E_{p}} & 0\\ 0 & 0 & \frac{1}{G_{p}} \end{array}\right]\left\{\begin{array}{c} T_{1}\\ T_{2}\\ T_{6} \end{array}\right\};$$

Hence, the equivalent parameter of PZT *a* can be expressed as follows:

(4) $$\left\{\begin{array}{c} E_{p}^{a}=\frac{1}{S_{11}^{E}}\\ v_{p}^{a}=-\frac{S_{12}^{E}}{S_{11}^{E}} \end{array}\right.$$

Hence, the equivalent parameter of PZT *a* can be expressed as follows:

(5) $$I_{a}=-A_{s}D_{s}^{a}s$$

where *s* = *iω* denotes the Laplacian operator; and *A_s_* is the area of the PZT’s electrodes. The correlation between voltage *V*_1_ and current *I_a_* is expressed as follows:

(6) $$V_{1}=-I_{a}Z_{1}=A_{s}D_{s}^{a}Z_{1}s$$

The capacitance’s impedance value, *Z*_1_, in the charge amplifier is given by *Z*_1_ = 1/(*C*_1_*s*). By solving Equations (2) and (6), the relationship between the internal mechanical strain $S_{1}^{a}$, $S_{2}^{a}$ and voltage *V*_1_ is as follows:

(7) $$V_{1}=\frac{A_{s}d_{31}Z_{1}s}{S_{11}^{E}S_{12}^{E}}(S_{1}^{a}+S_{2}^{a})=G(S_{1}^{a}+S_{2}^{a})$$

Herein, $G=A_{s}d_{31}Z_{1}s/S_{11}^{E}S_{12}^{E}$. From the above equation, it is evident that the voltage *V*_1_ is directly proportional to strains $S_{1}^{a}$ and $S_{2}^{a}$ with a constant ratio. Hence, *V*_1_ serves as an ideal sensor for strain in PZT *a*. At the subwavelength scale, the strain across PZT *a* and *b* can be approximated as uniform and reciprocally constrained:

(8) $$S_{1}^{a}=-S_{1}^{b};\;S_{2}^{a}=-S_{2}^{b}$$

The output voltage *V_out_* and current *I_b_* of PZT *b* satisfy the following relationship:

(9) $$\left\{\begin{array}{l} V_{out}=E_{3}^{b}h_{p}\\ I_{b}=A_{s}D_{3}^{b}s=\frac{\alpha V_{1}-V_{out}}{Z_{2}} \end{array}\right.$$

where *α* denotes *C_p_*/*C*_1_, and *Z*_2_ signifies the impedance of the circuit implemented by the microcontroller. Employing Equation (2), the constitutive equation for PZT *b* can be reformulated as follows:

(10) $$\left\{\begin{array}{l} S_{1}^{b}+S_{2}^{b}=(T_{1}^{b}+T_{2}^{b})(S_{1}^{b}+S_{2}^{b})+2d_{31}E_{3}^{b}\\ D_{3}^{b}=d_{31}(T_{1}^{b}+T_{2}^{b})+\varepsilon_{33}^{T}E_{3}^{b} \end{array}\right.$$

Subtracting stresses $T_{1}^{a}$ and $T_{2}^{a}$ from the above equation yields the following:

(11) $$\frac{S_{1}^{b}+S_{2}^{b}}{S_{11}^{E}+S_{12}^{E}}=\frac{D_{3}^{b}}{d_{31}}-\frac{\varepsilon_{33}^{T}}{d_{31}}E_{3}^{b}+\frac{2d_{31}}{S_{11}^{E}+S_{12}^{E}}E_{3}^{b}$$

Merging Equations (7), (9), and (11) yields the relationship between *V*_1_ and *V_out_*:

(12) $$V_{1}\left(\frac{Z_{2}}{Z_{1}}+\alpha \right)=\frac{V_{out}(h_{p}-A_{s}d_{31}Z_{2}s)}{h_{p}}\left(\frac{2d_{31}}{S_{11}^{E}+S_{12}^{E}}-\frac{\varepsilon_{33}^{T}}{d_{31}}\right)$$

Next, a parameter is defined:

(13) $$F=\frac{A_{s}d_{31}Z_{2}s}{h_{p}}\left(\frac{2d_{31}}{S_{11}^{E}+S_{12}^{E}}-\frac{\varepsilon_{33}^{T}}{d_{31}}\right)$$

Then, Equation (12) can be expressed as follows:

(14) $$\frac{V_{out}}{V_{1}}=\frac{Z_{2}/Z_{1}+\alpha }{1-FZ_{2}s}=H(s)$$

Combining Equations (2), (8) and (11), the constitutive equation of PZT *b* is expressed as follows:

(15) $$\left\{\begin{array}{l} S_{1}^{b}=S_{11}^{E}T_{1}+S_{12}^{E}T_{2}-\frac{d_{31}GH(s)}{h_{p}}(S_{1}^{b}+S_{2}^{b})\\ S_{2}^{b}=S_{12}^{E}T_{1}+S_{11}^{E}T_{2}-\frac{d_{31}GH(s)}{h_{p}}(S_{1}^{b}+S_{2}^{b})\\ S_{6}^{b}=2\left(S_{11}^{E}-S_{12}^{E}\right)T_{6} \end{array}\right.$$

Defining *A* = *d*_31_*GH*(*s*)/*h_p_*, the equation can be simplified as follows:

(16) $$\left\{\begin{array}{l} S_{1}^{b}=T_{1}\frac{S_{11}^{E}+A\left(S_{11}^{E}-S_{12}^{E}\right)}{1+2A}+T_{2}\frac{S_{12}^{E}+A\left(S_{12}^{E}-S_{11}^{E}\right)}{1+2A}\\ S_{2}^{b}=T_{1}\frac{S_{12}^{E}+A\left(S_{12}^{E}-S_{11}^{E}\right)}{1+2A}+T_{2}\frac{S_{11}^{E}+A\left(S_{11}^{E}-S_{12}^{E}\right)}{1+2A}\\ S_{6}^{b}=2\left(S_{11}^{E}-S_{12}^{E}\right)T_{6} \end{array}\right.$$

Similarly, by comparing with Equation (3), the equivalent elastic parameters of PZT *b* are derived as follows:

(17) $$\left\{\begin{array}{c} E_{p}^{b}=\frac{1+2A}{S_{11}^{E}+A\left(S_{11}^{E}-S_{12}^{E}\right)}\\ v_{p}^{b}=-\frac{S_{12}^{E}+A\left(S_{12}^{E}-S_{11}^{E}\right)}{S_{11}^{E}+A\left(S_{11}^{E}-S_{12}^{E}\right)} \end{array}\right.$$

Therefore, PZT *b* can be modeled as a homogeneous isotropic elastic medium, exhibiting frequency-dependent material properties, which can be computed using the aforementioned equations.

As illustrated in Figure 1, the amplification ratio of the amplifier circuit is *β*, governing the relationship between the output voltage *V_out_* and the controlled voltage *V*_2_, which satisfies the following:

(18) $$V_{out}=\beta V_{2}$$

The complete analog transfer function of the digital controller can be expressed as *H_DP_*(*s*) = *V*_2_/*V*_1_. By connecting Equations (14) and (18), we derive the following governing equation for the control system:

(19) $$H_{DP}(s)=\frac{H(s)}{\beta }=\frac{Z_{2}/Z_{1}+\alpha }{\beta (1-FZ_{2}s)}$$

## 3. Finite Element Modeling and Experimental Setup

**Figure 2:**
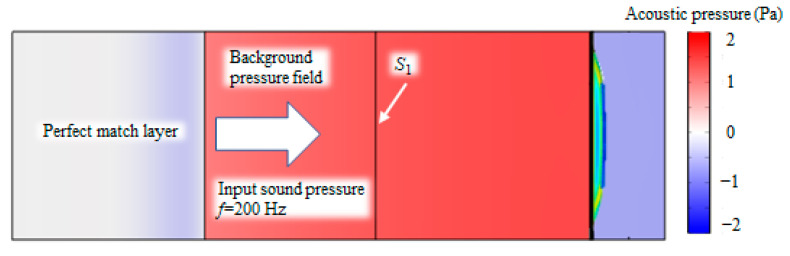
Finite element model and sound pressure distribution in tube.

To evaluate the acoustic absorption efficiency of the proposed metamaterial, a multi-physics coupling model was developed using COMSOL Multiphysics v6.1, integrating piezoelectricity, circuits, elastic structures, and acoustics. Figure 2 illustrates the finite element model for an incident wave frequency of 200 Hz and amplitude of 1 Pa, along with the corresponding sound pressure distribution. The simulation is driven by a background pressure field generating a plane wave propagating to the right. A perfect matching layer (PML) is positioned to the left of the pressure field to absorb reflected waves, while the remaining boundaries are modeled as hard sound field boundaries to simulate a rigid-walled waveguide, which is consistent with the acoustic impedance tube environment.

To determine the sound absorption coefficient of the proposed metamaterial, we calculated the incident sound power (*W_in_*) and reflected sound power (*W_ref_*) on plane *S*_1_:

(20) $$W_{in}=\int_{S_{1}}\frac{{P_{in}}^{2}}{2\rho_{0}c_{0}}dS$$

(21) $$W_{ref}=\int_{S_{1}}\frac{{P_{ref}}^{2}}{2\rho_{0}c_{0}}dS$$

The reflection coefficient is as follows:

(22) $$R=\frac{W_{ref}}{W_{in}}$$

Thus, the sound absorption coefficient can be obtained as follows:

(23) $$\alpha =1-{\left|R\right|}^{2}$$

Meanwhile, the acoustic impedance *Z_n_* of the metamaterial can be obtained by the following formula:

(24) $$Z_{n}=\frac{p}{v\cdot n}=R_{t}+X_{t}i$$

Here, **v** denotes the sound velocity near the metamaterial, and **n** is the unit vector in the normal direction. The real part of the acoustic impedance represents the acoustic resistance *R_t_*, while the imaginary part corresponds to the acoustic reactance *X_t_*. The sound absorption coefficient *α* of the metamaterial is directly related to its acoustic impedance. Consequently, the sound absorption coefficient can be expressed in terms of *R_t_* and *X_t_*:

(25) $$\alpha =\frac{4R_{t}}{{\left(1+R_{t}\right)}^{2}+{X_{t}}^{2}}$$

**Figure 3:**
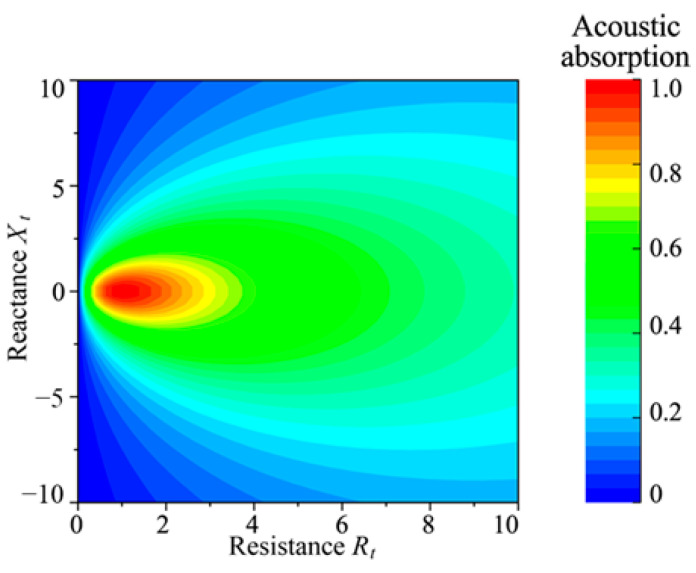
Graphs of the acoustic absorption coefficient *α* at varying values of *R_t_* and *X_t_*.

Based on Equation (25), the relationship between sound absorption coefficient *α*, acoustic resistance *R_t_*, and acoustic reactance *X_t_* can be obtained. Figure 3 presents the isocline graph of *α* against *R_t_* and *X_t_*, illustrating that achieving a high sound absorption coefficient requires satisfying two conditions: *R_t_* ≈ 1 and *X_t_* ≈ 0. Hence, to optimize sound absorption in the proposed metamaterial, the circuit parameters must be tuned to ensure *R_t_* and *X_t_* approach these target values as closely as possible.

**Figure 4:**
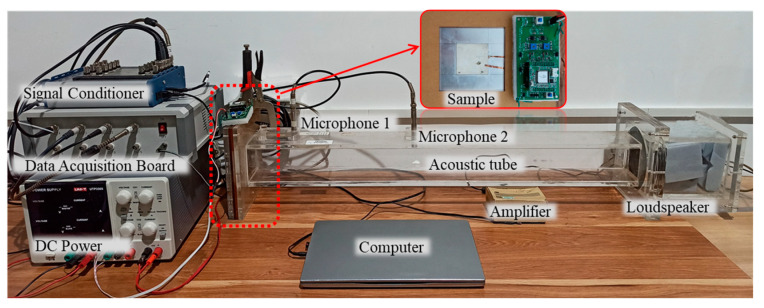
Photos of the experimental setup and the experimental sample.

Figure 4 illustrates the experimental setup, with the inset subgraph detailing the configuration of the composite sample. The sample features an aluminum membrane concentrically sandwiched between two PZT-5H piezoelectric patches, which were bonded using epoxy adhesive (Loctite 330) with a curing process at room temperature (about 25 °C) for 12 h. The geometric dimensions and material properties of both the aluminum membrane and PZT elements, as specified in Table 1 and Table 2, respectively, were provided by certified manufacturers: Hefei Xingfan Metal Materials Co., Ltd. (Hefei, China; aluminum components) and Baoding Hongsheng Acoustic Electronic Equipment Co., Ltd. (Baoding, China; piezoelectric elements). The positive and negative electrodes of the PZTs are connected to an external digital control circuit via copper foil outside the fixed frame. The low-cost digital control circuit integrates a Texas Instruments OPA454 precision operational amplifier (operational range: ±50 V) and an STM32F446 microprocessor equipped with two 12-bit DACs and multiple ADCs. The microprocessor was selected for its compatibility with low-frequency piezoelectric current control.

Sound absorption performance was evaluated using a custom-built acoustic impedance tube compliant with GB/T 18696.1-2004 (see [28] for design details). The tube, with a width matching the membrane dimension *l_Al_* = 0.08 m, incorporates two BSWA TECH MPA416-2 microphones spaced 350 mm apart on the specimen’s right side. A computer-generated white noise signal (100–500 Hz) is amplified by a power amplifier and used to drive a speaker at the right end of the tube, generating low-frequency noise. The signals captured by the microphones are converted into voltage signals by a signal conditioner (SKC Q882) and transmitted to a computer via a data acquisition card (National Instruments USB-4431). The sound absorption characteristics of the specimen are then calculated from the experimental data using custom-developed software.

## 4. Results and Discussion

### 4.1. “Resistance-Enhancement” Control

**Figure 5:**
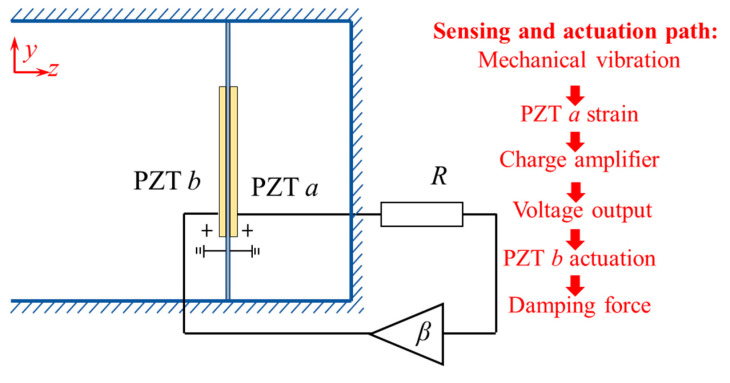
The resistance-enhancement control strategy.

The acoustic absorption performance of the proposed metamaterial is investigated. The digital control strategy enhances circuit efficiency, improving energy conversion between mechanical and electrical domains, thereby increasing the electromechanical coupling coefficient. Initially, the control strategy is configured to implement “resistance-enhancement” functionality as illustrated in Figure 5. Resistance in the circuit facilitates sound wave energy dissipation, and the integration of active enhancement control further amplifies this dissipation capability. As an example, the amplification factor is set to *β* = 10 and the resistance to *R* = 150 Ω.

**Figure 6:**
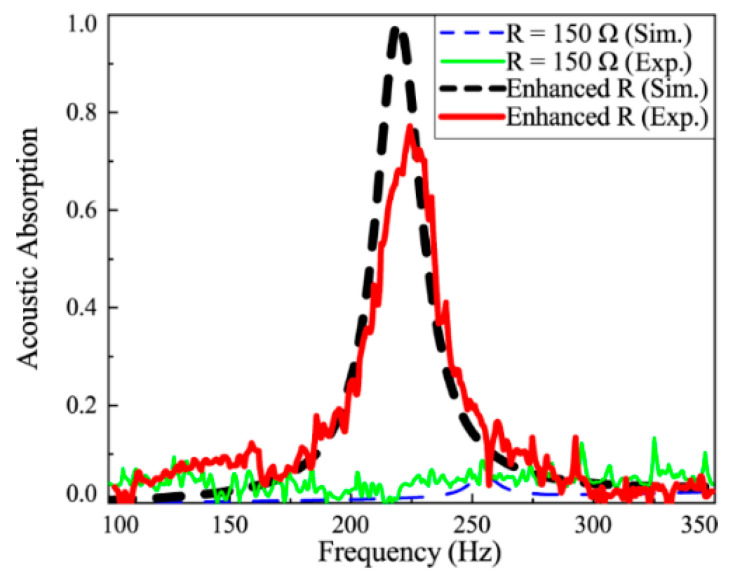
Sound absorption characteristics of the metamaterial with resistance-enhancement control strategy.

Figure 6 shows the absorption curve of the metamaterial under the “resistance-enhancement” control strategy. For comparison, the absorption curve of the metamaterial shunted with a passive resistor is also provided. The thick dashed and solid lines represent the simulation and experimental results for the actively controlled metamaterial, while the thin dashed and solid lines correspond to the passively shunted metamaterial. As shown in Figure 6, the absorption coefficient is nearly zero for the passively shunted metamaterial, indicating its ineffectiveness in absorbing and dissipating sound wave energy. In contrast, under the “resistance-enhancement” control strategy, the metamaterial achieves an absorption coefficient of 0.97 at approximately 224 Hz, demonstrating near-total absorption. It is worth noting that the resonance exploited in this strategy originates from the mechanical vibration modes of the piezoelectric membrane (natural frequencies: 224 Hz), not from electrical resonance in the shunt circuit. The digital controller dynamically adjusts the equivalent resistance to match the mechanical impedance of the membrane at these resonant frequencies, thereby maximizing energy dissipation. The close agreement between experimental and simulated results validates the effectiveness of the proposed control strategy.

The digital controller modulates the transfer function *H_DP_*(*s*) = *V*_2_/*V*_1_ according to Equations (15)–(17) to adjust the effective stiffness of PZT patches in real time. By adjusting parameters like the amplification factor *β* and circuit impedance Z_2_, the acoustic resistance is optimized; *R_t_* ≈ 1 and *X_t_* ≈ 0.

**Figure 7:**
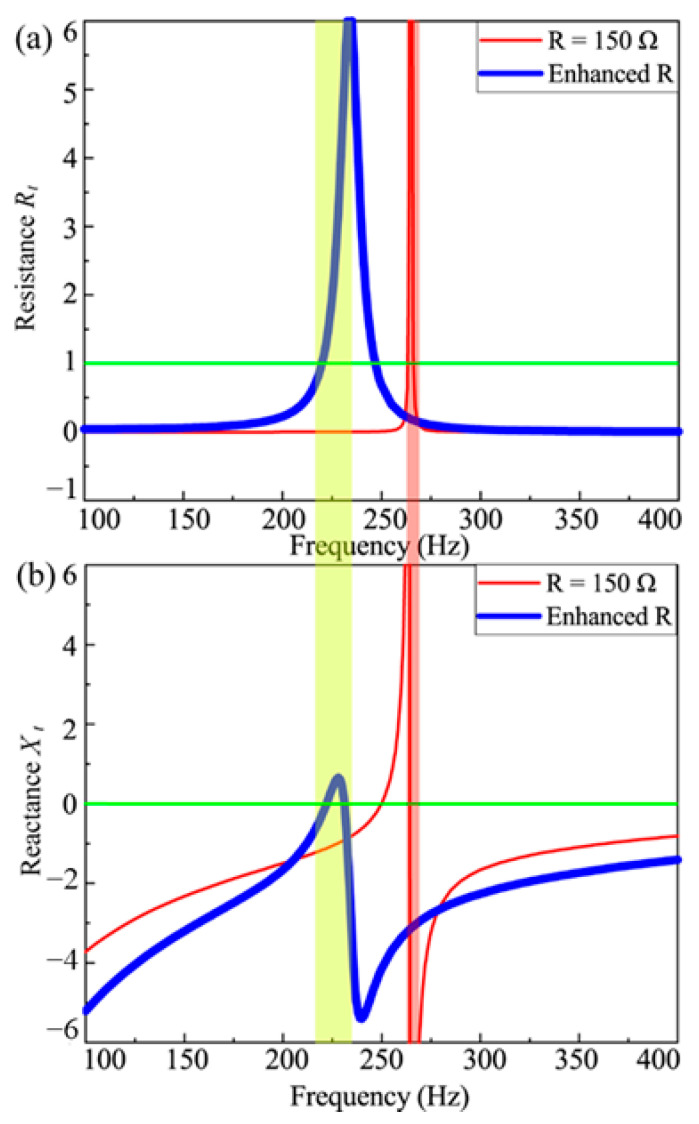
(**a**) The acoustic resistance and (**b**) the acoustic reactance curve of the metamaterial with resistance-enhancement control strategy.

The above discussion establishes the correlation between acoustic absorption and the acoustic resistance *R_t_* and reactance *X_t_*. Therefore, Equation (25) can be used to explain the exceptional acoustic absorption of the proposed metamaterial. Figure 7 illustrates the frequency-dependent variations in *R_t_* and *X_t_* for the “resistance-enhancement”-controlled metamaterial (thick line) and the passively shunted metamaterial (thin line).

As demonstrated in Figure 7a, the frequency response of *R_t_* for the passively shunted metamaterial exhibits a sharp peak near *R_t_* = 1, accompanied by *X_t_* approaching infinity. This configuration violates the high absorption condition (*R_t_* ≈ 1, *X_t_* ≈ 0), resulting in suboptimal sound absorption performance. In contrast, under the “resistance-enhancement” control strategy, the *R_t_* peak broadens significantly across the frequency range, and the associated *X_t_* curve becomes smoother. Near *R =* 1, *X_t_* approximates near zero, satisfying the high absorption requirement. Consequently, the actively controlled metamaterial achieves superior sound absorption performance.

It is noted that the frequency of near-perfect absorption coincides with the intrinsic resonance mode of the metamaterial. Near this resonance, the metamaterial undergoes significant deformation, amplifying the electromechanical coupling effect. The proposed control strategy enhances this coupling, improving energy conversion between mechanical and electrical domains, thereby increasing the effective resistance for sound wave dissipation. This mechanism enables the actively controlled metamaterial to achieve efficient sound absorption performance.

### 4.2. “Resonance-Enhancement” Control

**Figure 8:**
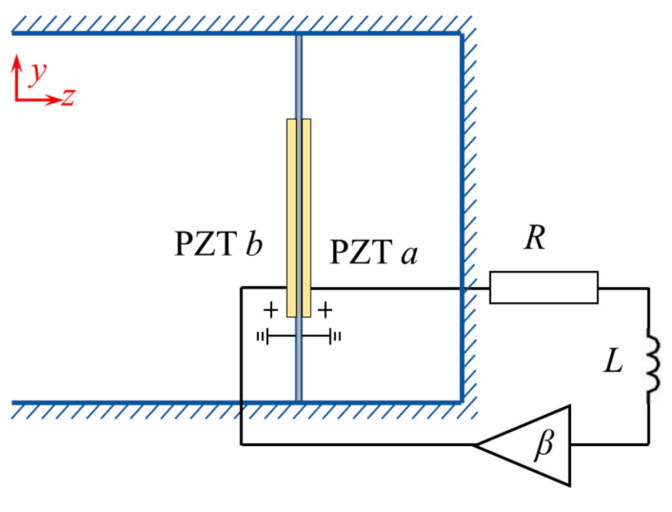
The resonance -enhancement control strategy.

In “resistance-enhancement” control, to achieve a broader absorption bandwidth, additional resonances can be integrated into the metamaterial, enabling multiple resonance amplifications within the target frequency range. In this section, we implement the “resonance-enhancement” control strategy using the controller illustrated in Figure 8. The resistor *R* remained at 150 Ω, while the inductance *L* was set to 1.5 H, and the amplification factor *β* was fixed at 10.

**Figure 9:**
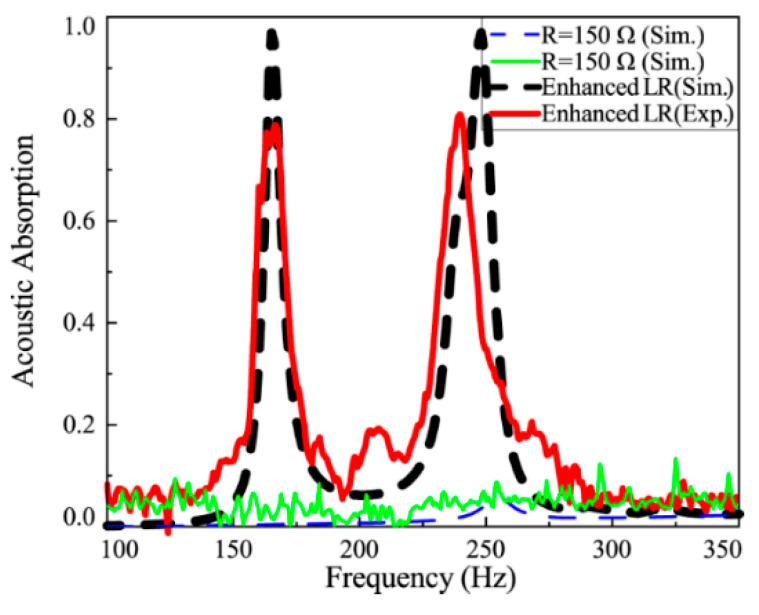
Sound absorption characteristics of the metamaterial with resonance-enhancement control strategy.

Figure 9 illustrates the sound absorption properties of the “resonance-enhancement”-controlled metamaterial compared to the passively shunted metamaterial. Two prominent absorption peaks are observed near 170 Hz and 248 Hz, with absorption coefficients exceeding 0.96, indicating near-complete absorption at these frequencies. The simulation and experimental results show excellent agreement, validating the effectiveness of the proposed control strategy.

**Figure 10:**
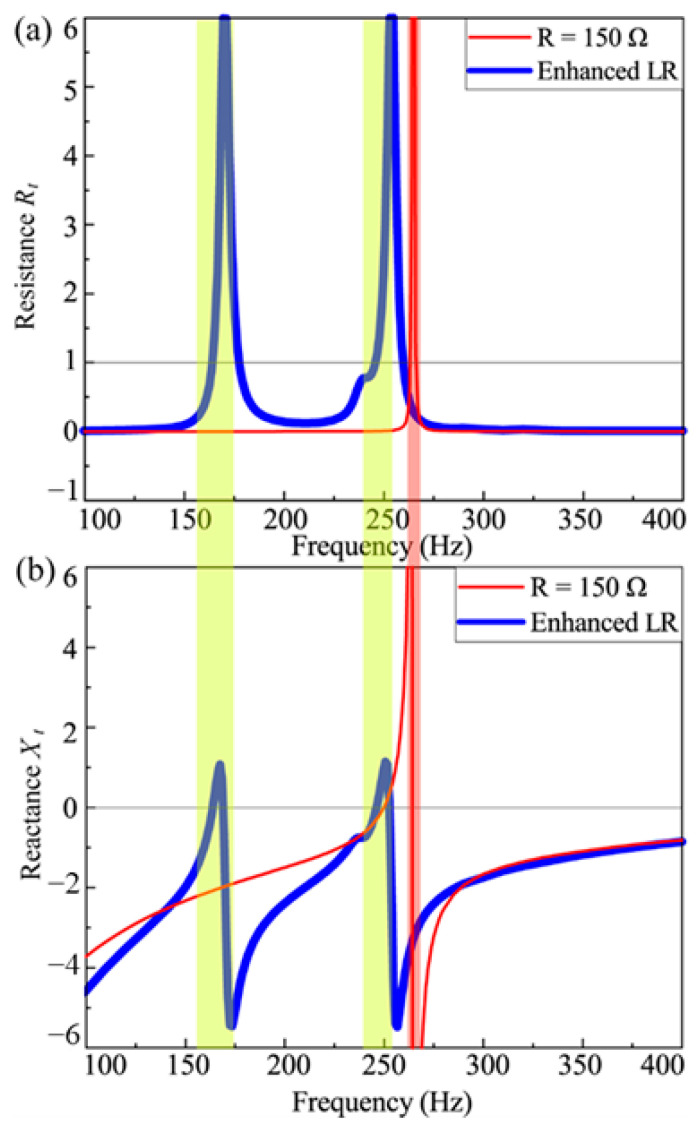
(**a**) The acoustic resistance and (**b**) the acoustic reactance curve of the metamaterial with resonance-enhancement control strategy.

In Figure 10, under “resonance-enhancement” control, the acoustic resistance *R_t_* exhibits two broad peaks in the frequency domain. One peak arises from the intrinsic resonance of the metamaterial, while the other is associated with the resonance generated by the control circuit. Notably, near *R_t_* = 1 at these peaks, the corresponding acoustic reactance *X_t_* becomes flat with values close to zero. This behavior indicates that the acoustic resistance and reactance at these frequencies satisfy the prerequisites for high sound absorption (*R_t_* ≈ 1, *X_t_* ≈ 0). Therefore, the metamaterial with the “resonance-enhancement” control strategy achieves a broader sound absorption bandwidth compared to other configurations, demonstrating its superior performance for broadband noise control.

Apparently, the proposed design exhibits superiority compared to the traditional design. Specially, passive designs (e.g., Helmholtz resonators [13,14] and membrane-based absorbers [18,19]) rely on fixed geometric resonances, making them ineffective for broadband or adaptive noise control. While passive metamaterials often require subwavelength thickness, our active design achieves comparable compactness (20 mm cavity depth) with a programmable frequency response.

## 5. Conclusions

This paper presents a digitally controlled membrane-type metamaterial for adjustable and broadband sound absorption. The proposed metamaterial consists of an aluminum membrane with two PZTs and an enclosed air cavity. The PZTs are shunted with a digital controlled circuit, enabling real-time tuning of the metamaterial’s acoustic properties. A piezoelectric-structure-acoustic coupling model is derived to evaluate the sound absorption capability of the metamaterial. Through theoretical analysis and experimental validation, we demonstrate the exceptional absorption performance of the metamaterial under both “resistance-enhancement” and “resonance-enhancement” control strategies. Compared with passive acoustic metamaterials and passive piezoelectric shunts, the proposed digital control strategy offers superior stability, real-time tunability, and broadband performance. The results indicate that the metamaterial achieves high sound absorption performance at subwavelength scales, particularly in the low-frequency range and near-resonance frequencies. The control strategy not only enhances electromechanical coupling efficiency but also enables real-time tuning of *R_t_* and *X_t_*, overcoming the fixed-frequency limitation of passive metamaterials and achieving broadband absorption. Furthermore, the adjustable transfer function of the digital control circuit enables real-time tuning of sound absorption performance without modifying metamaterial geometry, facilitating a broader absorption bandwidth. The compact design and real-time tunability of the proposed metamaterial make it particularly suitable for space-constrained environments requiring broadband noise control, such as aerospace cabins, urban infrastructure, and precision manufacturing facilities.

## Figures and Tables

**Table 1:** Geometric and physical parameters of membrane.

Parameters	Value
Length	$l_{\mathrm{Al}}=80\;\mathrm{mm}$
Thickness	$h_{\mathrm{Al}}=0.17\;\mathrm{mm}$
Density	$\rho_{\mathrm{Al}}=2700\;\mathrm{kg}\cdot m^{-3}$
Young’s modulus	$Y_{\mathrm{Al}}=69.5\;\mathrm{GPa}$
Poisson’s ratio	$v_{\mathrm{Al}}=0.3$

**Table 2:** Geometric and physical parameters of PZT.

Parameters	Value
Length	$l_{p}=40\;\mathrm{mm}$
Thickness	$h_{p}=0.25\;\mathrm{mm}$
Density	$\rho_{p}=7600\;\mathrm{kg}\cdot m^{-3}$
Compliance coefficient	$S_{11}^{E}=1.85\times 10^{-12}\;m^{2}\cdot N^{-1}$ $S_{12}^{E}=4.72\times 10^{-12}\;m^{2}\cdot N^{-1}$
Piezoelectric strain constant	$d_{31}=-101\times 10^{-12}\;C\cdot N^{-1}$
Dielectric constant	$\varepsilon_{33}^{T}=1.8\times 10^{-8}\;F\cdot m^{-1}$

## Data Availability

Data are contained within the article.

## References

[1] Tao Y., Ren M., Zhang H., Peijs T. (2021). Recent Progress in Acoustic Materials and Noise Control Strategies—A Review. Appl. Mater. Today.

[2] Yang M., Sheng P. (2017). Sound Absorption Structures: From Porous Media to Acoustic Metamaterials. Annu. Rev. Mater. Res..

[3] Zhang X., Qu Z., Wang H. (2020). Engineering Acoustic Metamaterials for Sound Absorption: From Uniform to Gradient Structures. iScience.

[4] Ji G., Huber J. (2022). Recent Progress in Acoustic Metamaterials and Active Piezoelectric Acoustic Metamaterials—A Review. Appl. Mater. Today.

[5] Qu S., Sheng P. (2022). Microwave and Acoustic Absorption Metamaterials. Phys. Rev. Appl..

[6] Cheng Y., Zhou C., Yuan B.G., Wu D.J., Wei Q., Liu X.J. (2015). Ultra-Sparse Metasurface for High Reflection of Low-Frequency Sound Based on Artificial Mie Resonances. Nat. Mater..

[7] Li Y., Assouar B.M. (2016). Acoustic Metasurface-Based Perfect Absorber with Deep Subwavelength Thickness. Appl. Phys. Lett..

[8] Long H., Shao C., Cheng Y., Tao J., Liu X. (2021). High Absorption Asymmetry Enabled by a Deep-Subwavelength Ventilated Sound Absorber. Appl. Phys. Lett..

[9] Liu X., Duan M., Liu M., Xin F., Zhang C. (2021). Acoustic Labyrinthine Porous Metamaterials for Subwavelength Low-Frequency Sound Absorption. J. Appl. Phys..

[10] Lemkalli B., Fellah Z.E.A., Guenneau S., Lamzoud K., Mir A., Achaoui Y. (2023). Bi-Functional Metamaterial Based on Helmholtz Resonators for Sound and Heat Insulation. arXiv.

[11] Ma K., Tan T., Yan Z., Liu F., Liao W.-H., Zhang W. (2021). Metamaterial and Helmholtz Coupled Resonator for High-Density Acoustic Energy Harvesting. Nano Energy.

[12] Ramos D., Godinho L., Amado-Mendes P., Mareze P. (2021). Experimental and Numerical Investigations of Ventilated Acoustic Metamaterial Based In-Parallel Arrangement of Helmholtz Resonator for Façade Screen. INTER-NOISE NOISE-CON Congr. Conf. Proc..

[13] Romero-García V., Jiménez N., Theocharis G., Achilleos V., Merkel A., Richoux O., Tournat V., Groby J.-P., Pagneux V. (2020). Design of Acoustic Metamaterials Made of Helmholtz Resonators for Perfect Absorption by Using the Complex Frequency Plane. Comptes Rendus Phys..

[14] Papadakis N.M., Stavroulakis G.E. (2023). Tunable Helmholtz Resonators Using Multiple Necks. Micromachines.

[15] Chen A., Yang Z., Zhao X., Anderson S., Zhang X. (2023). Composite Acoustic Metamaterial for Broadband Low-Frequency Acoustic Attenuation. Phys. Rev. Appl..

[16] Jung J., Park H., Park J., Chang T., Shin J. (2020). Broadband Metamaterials and Metasurfaces: A Review from the Perspectives of Materials and Devices. Nanophotonics.

[17] Ma G., Sheng P. (2016). Acoustic Metamaterials: From Local Resonances to Broad Horizons. Sci. Adv..

[18] Mei J., Ma G., Yang M., Yang Z., Wen W., Sheng P. (2012). Dark Acoustic Metamaterials as Super Absorbers for Low-Frequency Sound. Nat. Commun..

[19] Huang H., Cao E., Zhao M., Alamri S., Li B. (2021). Spider Web-Inspired Lightweight Membrane-Type Acoustic Metamaterials for Broadband Low-Frequency Sound Isolation. Polymers.

[20] Xu J., Yang R., Fan Y., Fu Q., Zhang F. (2021). A Review of Tunable Electromagnetic Metamaterials With Anisotropic Liquid Crystals. Front. Phys..

[21] Wang Y.-F., Wang Y.-Z., Wu B., Chen W., Wang Y.-S. (2020). Tunable and Active Phononic Crystals and Metamaterials. Appl. Mech. Rev..

[22] Cheer J., Hook K., Daley S. (2021). Active Feedforward Control of Flexural Waves in an Acoustic Black Hole Terminated Beam. Smart Mater. Struct..

[23] Danawe H., Tol S. (2023). Electro-Momentum Coupling Tailored in Piezoelectric Metamaterials with Resonant Shunts. APL Mater..

[24] Jian Y., Hu G., Tang L., Shen Y., Zhan Y., Aw K. (2023). Adaptive Piezoelectric Metamaterial Beam: Autonomous Attenuation Zone Adjustment in Complex Vibration Environments. Smart Mater. Struct..

[25] Liao Y., Chen Y., Huang G., Zhou X. (2017). Broadband Low-Frequency Sound Isolation by Lightweight Adaptive Metamaterials. J. Appl. Phys..

[26] Zhang H., Wen J., Xiao Y., Wang G., Wen X. (2015). Sound Transmission Loss of Metamaterial Thin Plates with Periodic Subwavelength Arrays of Shunted Piezoelectric Patches. J. Sound Vib..

[27] Zhang H., Xiao Y., Wen J., Yu D., Wen X. (2016). Ultra-Thin Smart Acoustic Metasurface for Low-Frequency Sound Insulation. Appl. Phys. Lett..

[28] Zhang X., Chen F., Chen Z., Wang G. (2018). Membrane-Type Smart Metamaterials for Multi-Modal Sound Insulation. J. Acoust. Soc. Am..

[29] Liao Y., Zhou X., Chen Y., Huang G. (2018). Adaptive Metamaterials for Broadband Sound Absorption at Low Frequencies. Smart Mater. Struct..

[30] Berardengo M., Manzoni S., Thomas O., Vanali M. (2018). Piezoelectric Resonant Shunt Enhancement by Negative Capacitances: Optimisation, Performance and Resonance Cancellation. J. Intell. Mater. Syst. Struct..

[31] Chen Y.Y., Huang G.L., Sun C.T. (2014). Band Gap Control in an Active Elastic Metamaterial With Negative Capacitance Piezoelectric Shunting. J. Vib. Acoust.

[32] Chen S., Wang G., Song Y. (2016). Low-Frequency Vibration Isolation in Sandwich Plates by Piezoelectric Shunting Arrays. Smart Mater. Struct..

[33] Wang G., Chen S. (2016). Large Low-Frequency Vibration Attenuation Induced by Arrays of Piezoelectric Patches Shunted with Amplifier–Resonator Feedback Circuits. Smart Mater. Struct..

[34] Wang G., Cheng J., Chen J. (2017). Multi-Resonant Piezoelectric Shunting Induced by Digital Controllers for Subwavelength Elastic Wave Attenuation in Smart Metamaterial. Smart Mater. Struct..

[35] Alshaqaq M., Sugino C., Erturk A. (2023). Digital Programming of Reciprocity Breaking in Resonant Piezoelectric Metamaterials. Phys. Rev. Res..

[36] Li X., Chen Y., Hu G., Huang G. (2018). A Self-Adaptive Metamaterial Beam with Digitally Controlled Resonators for Subwavelength Broadband Flexural Wave Attenuation. Smart Mater. Struct..

[37] Zhang X., Yu H., He Z., Huang G., Chen Y., Wang G. (2021). A Metamaterial Beam with Inverse Nonlinearity for Broadband Micro-Vibration Attenuation. Mech. Syst. Signal Process..

[38] Luo Z., Chen M.Z., Wang Z.X., Zhou L., Li Y.B., Cheng Q., Ma H.F., Cui T.J. (2019). Digital Nonlinear Metasurface with Customizable Nonreciprocity. Adv. Funct. Mater..

[39] Yi K., Matten G., Ouisse M., Sadoulet-Reboul E., Collet M., Chevallier G. (2020). Programmable Metamaterials with Digital Synthetic Impedance Circuits for Vibration Control. Smart Mater. Struct..

[40] Yan B., Wang K., Hu Z., Wu C., Zhang X. (2017). Shunt Damping Vibration Control Technology: A Review. Appl. Sci..

[41] Marakakis K., Tairidis G.K., Koutsianitis P., Stavroulakis G.E. (2019). Shunt Piezoelectric Systems for Noise and Vibration Control: A Review. Front. Built Environ..

[42] Zheng Y., Qu Y., Dai S., Chen B., Mao J. (2024). Mitigating Vibration and Sound Radiation with a Digital Piezoelectric Meta-Shell in Heavy Fluids. J. Sound Vib..

